# Correction: Drying-induced back flow of colloidal suspensions confined in thin unidirectional drying cells

**DOI:** 10.1039/d2ra90061k

**Published:** 2022-06-13

**Authors:** Kai Inoue, Susumu Inasawa

**Affiliations:** Graduate School of Bio-Applications and Systems Engineering, Tokyo University of Agriculture and Technology 2-24-16 Nakacho, Koganei Tokyo 184-8588 Japan inasawa@cc.tuat.ac.jp +81-42-388-7798 +81-42-388-7105; Department of Chemical Engineering, Tokyo University of Agriculture and Technology 2-24-16 Nakacho, Koganei Tokyo 184-8588 Japan

## Abstract

Correction for ‘Drying-induced back flow of colloidal suspensions confined in thin unidirectional drying cells’ by Kai Inoue *et al.*, *RSC Adv.*, 2020, **10**, 15763–15768, https://doi.org/10.1039/D0RA02837A.

The authors regret that an incorrect version of Fig. 3 was included in the original article. The transverse axes in Fig. 3e and its inset were incorrectly displayed. The correct version of Fig. 3 is presented below. The correction does not change any description, results or conclusions in the original article.

**Fig. 3:**
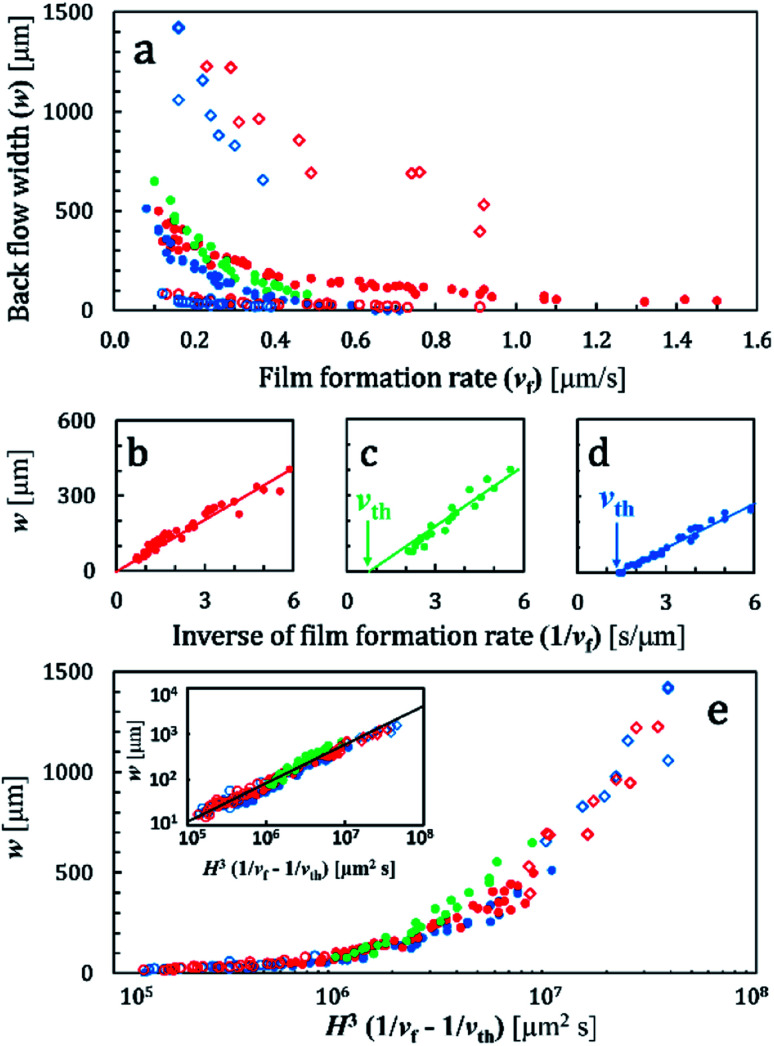
(a) Width of the back-flow region (*w*) and film growth rate (*v*_f_). Conditions (*d*, *φ*_w_, *H*) = (45 nm, 20 wt%, 100 μm) for red solid circles, (65 nm, 20 wt%, 100 μm) for green solid circles, (110 nm, 15 wt%, 100 μm) for blue solid circles, (45 nm, 20 wt%, 50 μm) for red open circles, (45 nm, 20 wt%, 200 μm) for red open squares, (110 nm, 15 wt%, 50 μm) for blue open circles, and (110 nm, 15 wt%, 200 μm) for blue open squares. Data of red, blue, and green solid circles in (a) are replotted by using *v*_f_^−1^ in (b)–(d). The solid line in (b)–(d) show a linear fitting result for the data for *v*_f_^−1^ < 6 [s μm^−1^]. (e) All data in (a) were plotted by using *H*^3^/(1/*v*_f_ − 1/*v*_th_). The inset in (e) shows the same data in a log–log plot. The solid line in the inset shows a slope of 0.85. The same symbols as in (a) are used in (b)–(e).

The Royal Society of Chemistry apologises for these errors and any consequent inconvenience to authors and readers.

## Supplementary Material

